# Organic Chemistry in Its Applications to Agriculture and Physiology

**Published:** 1843-10-01

**Authors:** 


I. ORGANIC CHEMISTRY, IN ITS APPLICATIONS TO AGRICULTURE AND PHYSIOLOGY. By *Justus Liebig*, M.D. &c. Edited by *Lyon Playfair*, Ph. D. 8vo. pp. 384. London: Taylor and Walton.

II. ELEMENTS OF AGRICULTURAL CHEMISTRY AND GEOLOGY. By *J. F. W. Johnston*, F.R.S. 12mo. pp. 250. Blackwood,

IT needs not, we should think, any argument to prove that it is absolutely necessary for medical men—if they expect to maintain that position in educated society which their profession may justly challenge, and which moreover all classes seem very willing to concede—to keep themselves up to the level of the existing state of science, especially of those branches of it which more immediately appertain to the general course of their studies. Chemistry is one of these, and a most important one it is; Botany, and indeed all natural history, is another; comparative Anatomy and Pathology (veterinary medicine) is a third; Geology and Meteorology are a fourth. A competent knowledge of these pursuits—besides throwing a dignified grace around the character of every man—will tend to correct many errors into which the unceasing occupation in the daily duties of his profession is apt to lead the practising physician; it will accustom him to take broad and comprehensive views on every question that is submitted to his notice, by enabling him to compare a multitude of facts with each other, and by teaching him to observe the mutual dependence and reciprocal action of the different objects in nature, and of the varied, though harmonious, agencies that are continually at work throughout its several kingdoms.

The great discoveries of medical science have all been made by men who were accomplished philosophers as well as eminent practitioners: we have only to point to those of *Harvey*, *John Hunter*, and *Jenner*. It is a very vulgar mistake to suppose that a due attention to matters of science, or even to the accomplishments of literature, is at all incompatible with a most assiduous and successful pursuit of active practice. It is far otherwise; the mind requires to be refreshed and enlarged by diversity of occupation to enable it to persevere with energy in its accustomed duties; and he, we may be assured, will generally make the best practical man, who can withdraw himself at intervals from the toil and turmoil of his daily occupations, and find a grateful refuge amid the pursuits of an enlarged and varied philosophy.

Medicine, it should be remembered, is, strictly speaking, one of the branches of physical science, and deserves to be studied and examined quite independently of its practical application to the relief of suffering. There are laws, no doubt, which regulate the development and the diffusion of diseases, just as there are laws which preside over the various changes and fluctuations of the ocean and the atmosphere. Who can look back upon the strange march of the pestilential Cholera ten years ago—starting from the fervid fields of India, and stalking on with slow but steady march across the steppes of Tartary to Russia, Denmark, Prussia, France and Britain; then traversing the broad Atlantic, and, after striking terror into the inhabitants of the New World, returning once more to Europe to visit its southern shores—without feeling the truth of this position? And if this be the case with the Cholera, so it is with almost all epidemic and with many endemic diseases: their origin, their progress and their decline are, we have every reason to believe, owing to the operation of certain physical agencies which are at work throughout the whole system of the universe, and the discovery of which, (though they are but imperfectly appreciable by us,) may yet reward the labours of some future enquirers.

But let us not be led away by any general disquisition from the more immediate object of the present article, which is to take a rapid survey of the great general truths of Vegetable Chemistry, and to point out the most striking relations that exist between the two kingdoms of living nature, animal and vegetable beings—their mutual dependences, their reciprocal actions, the sources whence they derive their elementary constituents, and the changes which they are constantly producing in the inorganic world around them.

There is not a greater marvel in the whole range of physical science than the fact of the countless diversities in the form, structure and visible appearances of organised matter contrasted with the elemental simplicity of its constitution. When we learn that every part of every plant and of every animal—from the moss to the cedar, and from the monad to man—is made up of but a few simple ingredients, and that all the exquisite machinery of their framework is composed of, and ultimately resolvable into, a little carbon and hydrogen, oxygen and azote, along with some saline or earthy matter, we feel from the first moment the utter hopelessness of ever being able *fully* to understand how these things can be. But although a dark veil hangs over such mysteries, we shall not be without our reward, if we will submit patiently to enquire and attentively to examine: for certainly most wonderful are the truths that have already been discovered. How marvellous the one fact that it is from the very dust of the valley, and from the air which we breathe, that there are produced and sustained all the glorious mechanisms of animal and vegetable life!

Independently of, but nevertheless in beautiful accordance with, the disclosures of revealed Truth, one of the earliest facts that arrests our attention is, that the creation of vegetable must necessarily have preceded that of animal beings. The former are designed to prepare the way for the very existence and support of the latter. No animal, we have reason to believe, can derive nourishment from or live upon inorganic substances; these may serve indeed to appease the cravings of hunger, but they cannot afford any supply of nourishment to the creature. Plants are manifestly the intermediate link and bond of connection between the animal and the mineral kingdoms of nature. They are endowed with the power of transferring the brute elements around them into organised productions; they suck in water and absorb atmospheric air, and, after decomposing them, they recombine their constituents in various modes, and thus assimilate them with the very texture and framework of their own systems. At the same time they associate unto themselves a certain quantity of earthy matter from the ground from which they spring—different alkaline and metallic salts, which are not only necessary for their own complete development, but are afterwards destined to become essential ingredients of animal bodies. Whence, for example, is the earthy matter of the bones, the various salts that abound in the urine and other fluids, the iron that is always present in the blood, &c. to be derived, except from the food on which the animal lives? Now (thanks to the discoveries of vegetable chemistry) all these substances can be proved to exist in the constitution of plants; and these, as we shall afterwards shew, obtain them all from the soil on which they grow. We thus at once perceive the necessity of our extending our enquiries beyond any one department of Nature's works, if we hope to arrive at a philosophic knowledge of its phenomena. This holds true especially of Animal Physiology; no rational system of this beautiful science can be constructed without a continual reference to the organisation and functions of plants.

Many, we know, entertain a vague and undefined idea that a living body has some mysterious power of generating or creating within itself some, if not all, of these substances to which we have been alluding; and certainly, until chemical science had attained that degree of exactitude to which it has reached only within the present century, it was scarcely possible to form even a probable conjecture as to the manner in which they were obtained. But this uncertainty is now past; for we know by the most undeniable experiments on the one hand, that there is not an element which enters into the constitution of an animal body, that does not exist in plants; and on the other, that there is not an element in the latter that cannot be proved to be derived from the mineral or inorganic kingdom. How emphatically true then is it of every living thing, that "dust it is, and unto dust it shall return." The decay of one generation serves but to supply the elements for the development and growth of a succeeding one; there is a constant and ever recurring cycle of life and death, of formation and dissolution; what was active yesterday is to day returning to its primal elements; and the very dust, that we trod upon an hour ago, is already becoming incorporated with a living frame, and will ere long be instinct with vitality.

The first question we propose to examine is the very curious one as to the source from which plants derive the various elements that enter into their composition.

We begin with *Carbon*. This elementary substance, it is well known, enters very largely into the framework of all vegetable matter. Its proportion, however, varies very greatly in different substances; as much as from 20 up to 80 per cent. We shall not perhaps exceed the truth if we state, in round numbers, that nearly one-half of the whole vegetable matter in the world consists of carbon or pure charcoal—a startling and seemingly at first an inexplicable announcement. Whence is it all derived? Hitherto it has been generally supposed that by far the greatest portion of this most necessary ingredient is obtained from the ground; and the language in common use has tended much to give rise to, and to perpetuate, this opinion. We talk of the rich and nutritious properties of certain soils, just in the same manner as we do of the fattening and restorative qualities of certain articles of food and drink to animals.

But there is a mistake in all this; for it is now ascertained, beyond a doubt, that no matter already organised can become assimilated with the substance of a living plant, unless it (the organised matter) has been previously resolved into its elementary constituents. Plants, as we have already remarked, live essentially and invariably on inorganic materials; and, when rich vegetable or animal manure is supplied to them with the view of promoting their speedy or exuberant growth, such substances act not by yielding any direct supply of nourishment, but only by facilitating certain changes and chemical decompositions, which we shall endeavour to explain as we proceed.

The Carbon of plants is derived in part from the earth, but chiefly from the atmosphere. The well-known circumstance that many plants thrive admirably well in water, and others in the air itself, affixed but by a filament to another plant, is almost a sufficient proof in itself of the truth of this assertion.

Few persons ask themselves, how do such plants obtain the nutriment that is necessary for their continuance and growth; or what is the source from which they obtain the large amount of carbon that they must have for the formation of flowers, and leaves, and branches? Is it the water, or is it the air? That it is not the former, we need not say to any one acquainted with the elements of chemistry; and that it is the latter, we shall now endeavour to shew. It will be useful first to recal a few circumstances connected with the constitution of the atmosphere, before we proceed.

It is well-known that atmospheric air is almost uniformly the same in composition in all places, seasons, and circumstances: the air, that has been collected at the height of 15 or 18,000 feet, is found to be quite similar to that at the surface of the earth; and what was present in jars buried for 1,800 years in Pompeii, contained the same elements, and in the same proportion, as that of the country in the present day. That there must be some great and marvellous agency at work, by which the constant waste of its oxygen is supplied, and by which the no less constant supply of carbonic acid is withdrawn, is therefore quite obvious. Now the beautiful researches of late years have shewn that it is the vegetable world that acts as the great purifier of the atmosphere for the respiration of animals; and, on the other hand, that it is the very change effected by this function in animals which furnishes the great supply of food for the growth of plants.

Every cubic foot of oxygen, consumed in respiration, is supplied by an equal volume of carbonic acid; and vice versa; every volume of carbonic acid, decomposed by vegetables, is replaced by an equal volume of pure oxygen. Keeping these facts in remembrance, let us now proceed in our enquiry, and see whether the air *can* afford the necessary supply of carbon to the vegetable world.

This question, says M. *Liebig*, is very easily answered.

"It is known, that a column of air of 2216.66 lbs. weight, Hessian measure, rests upon every square Hessian foot of the surface of the earth; the diameter of the earth and its superficies are likewise known, so that the weight of the atmosphere can be calculated with the greatest exactness. The thousandth part of this is carbonic acid, which contains upwards of 27 per cent. carbon. By this calculation it can be shown, that the atmosphere contains 3,000 billion

Hessian lbs. of carbon; a quantity which amounts to more than the weight of all the plants, and of all the strata of mineral and brown coal, which exist upon the earth. This carbon is, therefore, more than adequate to all the purposes for which it is required. The quantity of carbon contained in sea-water, is proportionally still greater." 22.

We have not deemed it at all necessary to *prove* that plants have the power of decomposing carbonic acid; for the fact is so easily shown. We have only to put a few fresh leaves in water containing carbonic acid, and expose the vessel to the sun's rays. After a short time, all the gas will be found to have disappeared from the water; and, if the experiment has been conducted under a glass receiver filled with water, the oxygen gas may be collected for examination. Plants do not emit oxygen in water that is not impregnated with carbonic acid, or which contains any alkali that may protect the acid from decomposition.

The quantity of the one gas decomposed, and of the other evolved, is uniformly in proportion to the intensity of the solar light to which the plant is exposed, and to the length of time it remains so. Hence, as our author beautifully remarks—

"The proper, constant, and inexhaustible sources of oxygen gas are the tropics and warm climates, where a sky, seldom clouded, permits the glowing rays of the sun to shine upon an immeasurably luxuriant vegetation. The temperate and cold zones, where artificial warmth must replace deficient heat of the sun, produce, on the contrary, carbonic acid in superabundance, which is expended in the nutrition of the tropical plants. The same stream of air, which moves by the revolution of the earth from the equator to the poles, brings to us in its passage from the equator, the oxygen generated there, and carries away the carbonic acid formed during our winter." 24.

When the light of day ceases, the decomposition of the carbonic acid in the atmosphere by the vegetable world no longer goes on, and oxygen is, therefore, no longer emitted. Indeed, the very reverse of this seems then to take place; for plants are found actually to give out carbonic acid, and sometimes also at the same time to absorb oxygen, during the night. This curious fact has been long known to vegetable physiologists and to chemists; but we are not aware that any one has ever taken the same view of it as M. *Liebig* does. It has been generally imagined that this evolution of carbonic acid, and absorption of oxygen during night, by plants, was a true process of respiration in them, similar to that in animals, and like it, having for its result the separation of carbon from some of their constituents. But this idea is, according to our author, quite a mistake; the process in question being, in fact, much rather of a simply mechanical, than of a truly vital, nature. The following is the explanation he gives of it.

"The carbonic acid which has been absorbed by the leaves and by the roots, together with water, ceases to be decomposed on the departure of day-light; it is dissolved in the juices, which pervade all parts of the plant, and escapes every moment through the leaves, in quantity corresponding to that of the water, which evaporates." 32.

.. .. .. .. .. "A cotton wick, inclosed in a lamp, which contains a liquid saturated with carbonic acid, acts exactly in the same manner as a living plant in the night. Water and carbonic acid are sucked up by capillary attraction, and both evaporate from the exterior part of the wick."* 33.

So much for the cause of carbonic acid being evolved in the dark; the process seems to be, so to speak, a mere act of evaporation, and has nothing to do either with the respiration or with the nutrition of the plant.

But then it may be asked,—admitting this manner of interpreting the disengagement of carbonic acid during the withdrawal of the solar light, how do you account for the absorption of oxygen at the same time? M. *Liebig* considers this process to be the result of a genuine chemical action between the gas in question, and certain of the organic constituents of the leaves and other parts of the plant—an action, he says, that is quite independent of life, as it goes on in a dead plant, exactly as in a living one. Whenever they (the leaves, &c.) contain either volatile oily matter which has a tendency to change into resin, or some other compound in which nitrogen is present, (as in nut-galls), it is found that the absorption of oxygen is much more active than under any other circumstances. The following paragraph is so interesting, and withal so full of curious instruction, that we must give it without abridgement:—

"The correctness of these inferences has been distinctly proved by the observations of *De Saussure;* for, whilst the tasteless leaves of the *Agave Americana* absorb only 0.3 of their volume of oxygen, in the dark, during 24 hours, the leaves of the *Pinus Abies,* which contain volatile and resinous oils, absorb 10 times, those of the *Quercus Robur* containing tannic acid 14 times, and the balmy leaves of the *Populus Alba* 21 times that quantity. This chemical action is shown, very plainly, also in the leaves of the *Cotyledon calycinum,* the *Cacalia ficoides* and others; for they are sour like sorrel in the morning, tasteless at noon, and bitter in the evening. The formation of acids is effected during the night, by a true process of oxidation: these are deprived of their acid properties during the day and evening, and are changed, by separation of a part of their oxygen, into compounds containing oxygen and hydrogen, either in the same proportions as in water, or even with an excess of hydrogen, which is the composition of all tasteless and bitter substances." 29.

If this view of the subjeet be the correct one, (and certainly it seems more than probable,) we at once perceive that the changes which plants produce in the surrounding air,—viz. the evolution of carbonic acid, and the absorption of oxygen—during exclusion from light, are almost quite independent of each other. For, while one seems to be the result of the evaporation of the superabundant juices, the other may be attributed to a mere chemical affinity between a gaseous element, viz. oxygen, and certain matters of vegetable origin, more especially volatile oils, tannin, &c. As a matter of course, we must not at once adopt the new and very ingenious views of the celebrated Professor of Giessen, until they have been tested by other enquirers, and their probability is more firmly established.

---

* Whether we adopt this interpretation or not, it is sufficiently obvious that it must be decidedly unwholesome to keep plants in a bed-room during the night. There is certainly no such objection, (but rather the very reverse,) to having them there during the day, provided they are kept in a part of the room where they are freely exposed to the light.

We do not profess to be very scientific chemists or vegetable physiologists ourselves, nor wish our readers to be hasty in admitting the truth of every novelty, however ingenious; but this one thing we would say; there is an *à priori* stamp of probability about the work of M. *Liebig* that strongly disposes us to admit the general truth of its positions, without waiting for further confirmation—and for nearly the same reason that we can often predict of a portrait whether it be a good likeness or not, although we may not have seen the original.

In our own experience we have more than once *felt* the force of this consideration, from the first moment that we got a glimpse of some fine discovery; it carried a resistless conviction with it at once by its beauty, its simplicity, its comprehensiveness, and its power of explaining a number of detached and seemingly dissociated facts, by a simple and easily intelligible principle. On no occasion was all this more emphatically felt, than when we first read Sir *Charles Bell*'s papers on the Spinal and Respiratory Nerves in the Royal Transactions; and we must confess that we have experienced something akin to this upon our first perusal of the present work: *mais revenons à nos moutons.*

It is not from the atmosphere alone that plants derive the carbon which enters so largely into their composition. The earth, too, and the rain which falls upon the earth, are almost continually yielding a certain amount of supply—not, indeed, in the form of pure carbon, (for that cannot be absorbed by any plant,) but in that of carbonic acid, which is afterwards decomposed within the body of the plant.

To explain this part of our subject fully, it would be necessary to describe at some length the various changes that go on during the processes of Fermentation and Putrefaction. A few words must, however, suffice for our present purpose.

The decay of all vegetable matter is, in truth, neither more nor less than a slow process of combustion, by which the destructible parts of a plant, combining with the oxygen of the atmosphere, are gradually consumed. Hence the necessity that there is of a constant renewal of air for the continuance of this process. Unless this be the case, the decay will at once be interrupted. The presence of a certain degree of moisture is no less needful to the change; alkalis promote it, while acids retard it; and all antiseptic substances, such as sulphurous acid, the mercurial salts, empyreumatic oils, &c. arrest it altogether. When the woody fibre of plants has entirely lost the property of further decay, or in other words, when it no longer has any tendency to unite with the oxygen of the atmosphere and generate carbonic acid, it becomes a brown coaly-looking substance, to which the name of *mould* is applied, and which constitutes the principal part of all the strata of brown coal and of peat. With this brief explanation, our readers will be better able to appreciate all the interest of the following very instructive passage:—

"*Humus,* (*i. e.* woody fibre in a state of decay,) acts in the same manner in a soil permeable to air as in the air itself; it is a continued source of carbonic acid, which it emits very slowly. An atmosphere of carbonic acid, formed at the expense of the oxygen of the air, surrounds every particle of decaying humus. The cultivation of land, by tilling and loosening the soil, causes a free and unobstructed access of air. An atmosphere of carbonic acid is, therefore, contained in every fertile soil, and is the first and most important food for the young plants which grow in it.

"In Spring, when those organs of plants are absent which nature has appointed for the assumption of nourishment from the atmosphere, the component substance of the seeds is exclusively employed in the formation of the roots. Each new radicle fibril which a plant acquires may be regarded as constituting at the same time a mouth, a lung, and a stomach. The roots perform the functions of the leaves from the first moment of their formation; they extract from the soil their proper nutriment, namely, the carbonic acid generated by the humus.

"By loosening the soil which surrounds young plants, we favour the access of air, and the formation of carbonic acid; and on the other hand the quantity of their food is diminished by every difficulty which opposes the renewal of air. A plant itself effects this change of air at a certain period of its growth. The carbonic acid which protects the undecayed humus from further change, is absorbed and taken away by the fine fibres of the roots, and by the roots themselves; this is replaced by atmospheric air, by which process the decay is renewed, and a fresh portion of carbonic acid formed. A plant at this time receives its food, both by the roots, and by the organs above ground, and advances rapidly to maturity." 48.

At this part of his beautiful dissertation, M. *Liebig* alludes to an interesting fact well-known to all geologists, but of which no satisfactory explanation has hitherto, as far we know, been attempted to be given. The plants of the primeval world appear (from their remains found in the coal formation) to have consisted chiefly of gigantic mono-cotyledonous ferns, palms and reeds—plants to which nature has given the power, by means of an immense extension of their leaves, to dispense with any great supply of nourishment from the soil. It would seem, therefore, that their roots act but a very subordinate part in their growth. Now the curious fact is this, that while we find in the bowels of the earth vast quantities of fossilized leaves, stems, and fruit, scarcely any traces of the roots of these primæval trees have ever been discovered. What explanation shall we give of this? May it not be that—as there could be comparatively but little mould on the surface of the earth in these primæval days, and as there is every reason to believe that the solar light and heat were then unusually great—the nourishment of the plants, that covered the earth, was effected almost altogether by their leaves, and only in a very inferior degree, if at all, by their roots?

*Hydrogen.*—The proportion of this element is found to vary considerably in different parts and products of a plant. In *some*, as in lignin, sugar, gum, and starch, its proportion to that of the oxygen present is exactly that in which these two elements combine to form water; there is no *excess* either of the one or of the other.

In *other* vegetable matters, as in oils, resin, wax, &c. the proportion of hydrogen present exceeds that which is necessary to form water with the existing oxygen; while in a *third* set, as in all the vegetable acids for example, the oxygen is in excess, and the proportion of hydrogen is consequently deficient. There are exceptions, we believe, to the uniform accuracy of this ternary division of vegetable substances and productions; but for general purposes it is sufficiently correct.

That the Hydrogen of plants is derived from the decomposition of water, is admitted by all enquirers.

"The process of assimilation," says our author, "in its most simple form, consists in the extraction of hydrogen from water, and carbon from carbonic acid; in consequence of which, either all the oxygen of the water and carbonic acid is separated, as in the formation of caoutchouc, the volatile oils which contain no oxygen, and other similar substances, or only a part of it is exhaled." 66.

The influence of solar light, in aiding the decomposition of water and the exhalation of its oxygen, is apparent from the well-known facts, that, 1, in this country, when the Summer is unusually gloomy and cold, fruits never ripen thoroughly, but remain more or less sour, (in consequence of their acid matter not being converted into sugar); and 2, that a large proportion of tropical trees abound in oils, caoutchouc, and other substances, which contain very little if any oxygen. Such vegetable productions have little or no tendency to decay or putrefaction; and for this simple reason, that their chief constituent has a very weak disposition to combine with the oxygen of the atmosphere.

*Nitrogen.*—It was long supposed that this element was contained in very few substances of vegetable origin, and that its presence was confined almost exclusively to those of an animal nature, and was thus in some measure a mark of distinction between the two kingdoms of nature. It is now, however, well ascertained, that it is much more largely diffused through most plants than was imagined; and although, when estimated by its proportional weight, it forms but a very small part of their composition, it is (says M. *Liebig,*) never entirely absent; for, even when it does not absolutely enter into the composition of a particular part or organ of a plant, it is always to be found in the fluids pervading it. Of all vegetable substances, it is most abundant in albumen and gluten, and in all the organic bases or alkaloids, such as morphia, strychnine, &c.

Whence is the Nitrogen of plants derived? The opinions of M. *Liebig* on this subject are among the most interesting novelties of the work before us. He shews, in the first place, that there are many good reasons for believing that this element is never obtained from the atmosphere—at least directly, and in its simple uncompounded form. What then is the probable source of it?—this is the point we shall now endeavour to explain.

There is a substance very widely diffused throughout nature, capable of existing in a variety of conditions and of entering into a variety of combinations, having a strong affinity for carbonic acid, emitted from almost every body in a state of decomposition, very volatile and at the same time very soluble in many of its forms, present in the air around us, and in the rain that falls on the ground, and in the waters of the ocean; and of this substance, nitrogen (existing in a readily separable or loosely combined condition) is a large constituent. Need we add that this substance is Ammonia?

When we consider that there is a constant and unceasing evolution of it in the state of vapour, springing from the decomposition of animal matter at almost every point of the earth's surface, we may form some idea of the enormous aggregate quantity that must be diffused through the atmosphere.

"A generation of a thousand million men," says our eloquent author, "is renewed every thirty years: thousands of millions of animals cease to live, and are reproduced, in a much shorter period. Where is the nitrogen which they contained during life? There is no question which can be answered with more positive certainty. All animal bodies, during their decay, yield the nitrogen, which they contain, to the atmosphere, in the form of ammonia. Even in the bodies buried sixty feet under ground in the churchyard of the Eglise des Innocens, at Paris, all the nitrogen contained in the adipocire was in the state of ammonia. Ammonia is the simplest of all the compounds of nitrogen; and hydrogen is the element for which nitrogen possesses the most powerful affinity." 73.

As Ammonia and its volatile compounds are extremely soluble in water, its vapour in the atmosphere is speedily dissolved in the moisture diffused through it, and returns again to the earth in every shower of rain. Rain and snow water almost always contain a certain amount of carbonate of ammonia, varying much indeed in different seasons and in different localities. The quantity is greater in Summer than in the Spring or Winter, because the intervals of time between the showers in the former season are greater; and when several wet days occur together, the rain of the first day must contain more of it than that of the second. The presence of ammonia in rain is readily shewn by merely adding a little sulphuric or muriatic acid to it, and evaporating it nearly to dryness: the ammoniacal salt is detected by the peculiar pungent odour, exhaled upon adding a little powdered lime to the residue. The *softness* of rain water is owing to the presence of carbonate of ammonia in it.

That Ammonia exists pretty freely in many parts of certain plants is abundantly obvious. The juices of the maple and of the birch yield it readily; so do all vegetable extracts; as well as most flowers, leaves (especially those of the tobacco plant) and roots when distilled with water; also not a few fruits, as for example the unripe pulp of the almond and peach, &c.

It is from the Ammonia, obtained by the roots of plants in the manner we have now explained, that most of the Nitrogen that exists in so many of their products, as their gluten, albumen and alkaloid bases, is derived. Here we may observe that the nutritious quality of vegetable food is, in a great measure, proportionate to the quantity of azotised materials which it contains. A horse may indeed be kept alive by feeding it with potatoes, (which contain a very small quantity of nitrogen); but life thus supported is a gradual starvation; the animal increases neither in size nor strength, and sinks under every exertion. The quantity of rice that a Hindoo will consume often astonishes a European; but our astonishment ceases, when we know that this grain contains a very small proportion of azotised matter. The system is constantly requiring a certain supply, to compensate for the continual expenditure by the urinary secretion and in other ways; and the person is therefore obliged to make up by the quantity, for the unsatisfactory quality, of the food taken.

How far M. *Liebig* is correct in his theory that most of the nitrogen of plants (and therefore of animals too) is primarily derived in the state of ammonia, we cannot take upon ourselves to determine. Mr. *Johnston*, we observe, inclines to the opinion that the chief supply is rather from the *nitrates*, that are almost always present in the soil. The generation of Nitrico Acid seems to be continually going on, wherever vegetable matter is in a prcess of decay. It is produced also largely in some climates during thunder storms—every flash of lightening causing the union of the two elements of the atmosphere, along the line of its track. Certain it is that the presence of nitrate of potash or of soda in most soils contributes greatly to their fruitfulness, and we know that vegetation is always very luxuriant where these salts abound.

Whichever view we take of this question (and there is probably truth in both), we cannot deprive ourselves of the pleasure of extracting the following passage, as it will give our readers a good idea of M. *Liebig's* felicity of illustration.

"Let us picture to ourselves," says he, "the condition of a well-cultured farm, so large as to be independent of assistance from other quarters. On this extent of land, there is a certain quantity of nitrogen contained both in the corn and fruit which it produces, and in the men and animals which feed upon them, and also in their excrements. We shall suppose this quantity to be known. The land is cultivated without the importation of any foreign substance containing nitrogen. Now, the products of this farm must be exchanged every year for money, and other necessaries of life, for bodies therefore which contain no nitrogen. A certain proportion of nitrogen is exported with corn and cattle; and this exportation takes place every year, without the smallest compensation; yet after a given number of years, the quantity of nitrogen will be found to have increased. Whence, we may ask, comes this increase of nitrogen? The nitrogen in the excrements cannot reproduce itself, and the earth cannot yield it. Plants, and consequently animals, must, therefore, derive their nitrogen from the atmosphere." 72.

We can now better understand the reason why animal manure (being so rich in nitrogen) is so necessary for the successful cultivation of all the esculent grains, as well as of grass and other fodder for the use of cattle. The proportion of glutinous matter—the nutritious constituent—in wheat, &c. is found to vary very much according to the kind and quantity of the manure, with which the ground has been supplied. For example, it has been found that 100 parts of wheat grain on a soil, manured with cow-dung (a manure which contains the smallest quantity of nitrogen), afforded only 11·95 parts of gluten, and 64·34 parts of amylin or starch; whilst the same quantity of the grain, grown on a soil manured with human urine,* yielded as much as 35 per cent. of gluten.

Putrescent urine, it is well known, abounds in azotised ingredients in the form of ammoniacal salts—the urea being converted, under the influence of heat and moisture, almost exclusively into carbonate of ammonia.

* We need scarcely remind the reader that the kidneys are the great emunctories for the discharge of nitrogen from the body, and that every lesion therefore of their function is necessarily accompanied with some disturbance in the eliminating processes, that are continually going on throughout all parts of the animal economy.

The solid excrements of animals give out much less ammonia, and consequently contain much less nitrogen, than their urine ; and here it deserves to be noticed that the more perfectly that the food, more especially in the case of herbivorous animals, is digested and assimilated, the more completely is it deprived of its azotised ingredients, and therefore the less fertilising will be the ordure. There is so much curious and instructive information in the following extract that we must give it without abridgment.

"Liquid animal excrements, such as the urine with which the solid excrements are impregnated, contain the greatest part of their ammonia in the state of salts, in a form, therefore, in which it has completely lost its volatility when presented in this condition; not the smallest portion of the ammonia is lost to the plants; it is all dissolved by water, and imbibed by their roots. The evident influence of gypsum upon the growth of grasses—the striking fertility and luxuriance of a meadow upon which it is strewed—depends only upon its fixing in the soil the ammonia of the atmosphere, which would otherwise be volatilised with the water which evaporates. The carbonate of ammonia contained in rain-water is decomposed by gypsum, in precisely the same manner as in the manufacture of sal-ammoniac. Soluble sulphate of ammonia and carbonate of lime are formed; and this salt of ammonia possessing no volatility is consequently retained in the soil. All the gypsum gradually disappears, but its action upon the carbonate of ammonia continues as long as a trace of it exists." 87.

Before quitting this part of our subject, we may allude for a moment to the spontaneous generation of *nitre* or saltpetre in the soil of certain countries, more especially of the East Indies—from which, it is well known, our chief supply of this substance is obtained. The formation of the nitric acid seems to be the result of a slow combination of the nitrogen in the ammonia, abounding in the soil, with the oxygen of the atmosphere —a combination which is much promoted by the presence of alkaline bases, at the same time, in the former.* We have already alluded to the production of nitric acid in the atmosphere during thunder-storms; and certainly the frequency of these in the East Indies may, in some measure, account for the abundant formation of saltpetre on the surface of the ground in that country. In many parts of the Continent, it is prepared artificially from a mixture of common mould with animal and vegetable remains containing nitrogen : nitric acid is gradually generated, and unites with the potash, lime and magnesia that are usually present. By dissolving these salts in water, and precipitating the two earths by carbonate of potash, a solution of nitre is obtained. But this is a subject which we need not pursue ; suffice it merely to say the addition of the nitrates to certain soils has been found to increase their productiveness in a very remarkable degree.

Having now considered the primary and fundamental elements of vege-

---

* "Ammonia, by its transformation," says M. *Liebig*, "furnishes nitric acid to the tobacco plant, sunflower, *Chenopodium*, and *Borago officinalis*, when they grow in a soil completely free from nitre. Nitrates are necessary constituents of these plants, which thrive only when ammonia is present in large quantity, and when they are also subject to the influence of the direct rays of the sun—an influence necessary to effect the disengagement within their stem and leaves of the oxygen, which shall unite with the ammonia to form nitric acid." 82.

table substances, we are prepared to advance a step in our enquiry, and proceed to the examination of—

*The Mineral Constituents of Plants.*

From the preceding observations it will be remarked that all the elements, necessary for the support of animal and vegetable life, are contained in three substances, which are universally diffused throughout the atmosphere, viz. Carbonic acid, Water, and Ammonia. Now, it is a curious and most interesting fact that these are the very substances which are the ultimate products of the putrefaction and decay of organized matter. "All the innumerable products of vitality," to use the expressive language of our author, "resume after death the original form from which they sprung. And thus death—the complete dissolution of an existing generation—becomes the source of life for a new one."

Besides, however, the simple elements of oxygen, hydrogen, carbon, and, we may now add, nitrogen, there are certain solid mineral substances (usually of an alkaline, earthy, or metallic nature) which are present in almost all, if not in all, plants, and the presence of which is necessary to their proper growth, if not to their very existence. These saline matters vary a good deal, in kind as well as in quantity, in different tribes of plants. The ashes of all are found to contain an alkaline carbonate; and we may therefore assume that the presence of a certain amount of Potash or of Soda is absolutely requisite for the healthy condition of a vegetable.

The various kinds of grasses and reeds, and especially the tribe of the Equisetaceæ, contain in the outer parts of their leaves and stalks a large quantity of Silica in union with Potash.* If a due quantity of the latter substance be not constantly present in the soil, the crops of grass or of grain raised upon it will soon begin to fail, unless an artificial supply be furnished by strewing the surface with wood-ashes, or some other substance that can afford it.

The great fertility of the soil, that is formed by the disintegration of Lava and other volcanic matters, seems to be owing to the quantity of potash which it contains. The following extract affords another striking illustration of the same fact.

"The first colonists of Virginia found a country, the soil of which was similar to that mentioned above; harvests of wheat and tobacco were obtained for a century from one and the same field without the aid of manure, but now whole districts are converted into unfruitful pasture land, which without manure produces neither wheat nor tobacco. From every acre of this land, there were removed in the space of one hundred years 1200lbs. of alkalies in leaves, grain, and straw; it became unfruitful therefore, because it was deprived of every particle of alkali, which had been reduced to a soluble state, and because that which was rendered soluble again in the space of one year, was not sufficient to satisfy the demands of the plants. Almost all the cultivated land in Europe is

* The quantity of silicate of potash, that is removed from a meadow, in the form of hay, is very considerable. A curious illustration of this occurred some time ago near Heidelberg. A stack of hay was struck by lightning, and reduced to a mere vitreous mass. The substance, when first found, was supposed to be a Meteoric stone.

in this condition; fallow is the term applied to land left at rest for further disintegration. It is the greatest possible mistake to suppose that the temporary diminution of fertility in a soil is owing to the loss of humus; it is the mere consequence of the exhaustion of the alkalies." 149.

All our Cereal or corn plants, as well as many others, require, for the due development of their seeds, a supply also of phosphate of Magnesia* and of Ammonia. Now these salts, although present in most cultivated lands, are only found abundant in those soils where men and animals are dwelling together, and where consequently there can be a supply of proper manure. It has often been a subject of puzzling speculation to account for the well-known fact that the Cereal grains are never fully and completely developed, except in the neighbourhood of the habitations of men. The explanation is very simple: this tribe of plants requires a certain supply of particular saline matter (besides nitrogen), which is not readily obtained except in the manner now mentioned.† For the same reason, sea-plants will not thrive in an inland situation, unless indeed near salt-works, which may be several hundred miles distant from the sea; and the *Chenopodium* is always found near a dunghill, in order that it may find a supply of ammonia and of the nitrate salts. Oxalate of Lime is invariably present in lichens, and seems indeed to occupy the place of woody fibre, which is absent; while the oxydes of Iron and of Manganese, not to mention Sulphur in some of its forms of combination, and Phosphorus, are found in other tribes of the vegetable world.

We have now enumerated all, or nearly all, the inorganic mineral substances which are found in land plants; Iodine and Bromine exist, it is well known, in many marine vegetables. Let us now enquire what is the source of these substances, and in what manner plants obtain and incorporate them with their systems. Of course, the constant decay of vegetable matter itself, that is going on everywhere, restores to the soil no inconsiderable portion of the saline matter, which had been previously derived from it. But this will by no means account for the entire supply; and we must therefore look elsewhere for the store-house, from which it must all have primarily come. In this, as in every other, arrangement of the material world, there will be found to be a beautiful mechanism of alter-

* "Phosphate of magnesia in combination with ammonia is an invariable constituent of the seeds of all kinds of grasses. It is contained in the outer horny husk, and is introduced into bread along with the flour, and also into beer. The bran of flour contains the greatest quantity of it. It is this salt which forms large crystalline concretions, often amounting to several pounds in weight, in the *cæcum* of horses belonging to millers."

We do not now, with such a fact as this before us, require to be told whence the animal system derives its supply of phosphatic salts for the formation of the bones, and other parts of the body in which they exist.

† "The small quantity of phosphates, which the seeds of the lentils, beans and peas contain, must be the cause of their small value as articles of nourishment, since they surpass all other vegetable food in the quantity of nitrogen which enters into their composition. But as the component parts of the bones (phosphate of lime and magnesia) are absent, they satisfy the appetite without increasing the strength."

nating and reciprocal compensation, which proclaims at once the power and the wisdom of the Great Architect of all things.

The parent source, from which by far the largest supply of the saline matter of the soil, and consequently of its productions, is derived, is unquestionably the evaporation that is continually going on from the surface of the ocean. The salts, which most abound in sea-water, are the chlorides of Sodium, Potassium, and Magnesium, and the sulphate of soda: there is also a small proportion of the sulphate, and a still smaller of the carbonate, of Lime. It is from this last-named salt, although its quantity be so minute that its presence is scarcely appreciable in a pound of sea-water, that the marine Mollusca and coral animals derive all the materials for the formation of their coverings and habitations. Ammonia also is found in sea-water. *Marcet* has expressly told us that "when the solid saline residue obtained by evaporation is heated in a retort to redness, a sublimate of sal-ammoniac is obtained."

Thus, then, we find that the water of the ocean contains those very substances—viz. Carbonic Acid, Water, and Ammonia—which we have already shewn to be the sources from which plants derive their subsistence on the land. Such then being the case, we can have no difficulty in understanding how the weeds, that grow attached to rocks at the bottom of the ocean, obtain those elements which are essential to the very constitution of vegetable matter; and we have presented to our view a new illustration of the all-perfect adaptation of Nature's works to the varying circumstances in which they happen to be placed.

Having now pointed out the various constituent elements that enter into the composition of vegetable substances, and explained the sources from which these are derived, we shall occupy the rest of this article with what may be called the practical application of the subject, in reference more particularly to the diet and food of animals.

An obvious inference from many of the foregoing details may probably have suggested itself already to most of our readers, viz. that the products, generated by any plant, will vary considerably according to the circumstances in which it is placed, and the soil on which it grows; and for this very simple reason, that the different products contain very different proportions of the elementary vegetable constituents.

We have seen, for example, that the glutinous and albuminous parts of plants contain a larger proportion of Nitrogen than any other; and we are therefore prepared to expect that, unless a due supply of azotised materials be supplied to the plant during its growth, the formation of these vegetable substances will be but imperfect, and will be, to a greater or less extent, replaced by that of other substances in which nitrogen is less predominant: such are sugar, starch, oil, wax, or gum. Hence, as our author remarks, the mode of culture, employed for the purpose of procuring fine pliable straw for Florentine hats is the very opposite to that which must be adopted in order to produce a maximum of corn from the same plant. One peculiar method must be used for the production of nitrogen in the seeds; another for giving softness and pliancy to the straw, and another still, when we wish to give such strength and solidity to the stem as will enable it to bear the weight of heavy ears. In illustration of the same subject, we may state that the quantity of Fecula in potatoes increases, when the soil contains much decaying vegetable fibre; but decreases when it is manured with strong animal manure,—the potatoes acquiring, in the first instance, a mealy, and in the second, a soapy consistence. It is obvious, therefore, that the agriculturist must proceed in the culture of plants on very analogous principles to those which have been so satisfactorily adopted of late years in improving the breeds of different animals, according to the object which the farmer may have in view—whether this be to increase or diminish the quantity of fat, to improve the character of the hide, to lessen the size of the bones, and so forth. But as this subject scarcely comes within the reach of our enquiries—except indeed as illustrating the influence of external objects on animal life—we must pass it over for another, which is not only of very curious, but really of practical, interest to the medical man, viz. the relative value of different vegetable substances as food for men and animals, judging of them by their chemical constitution. We have already more than once alluded to the circumstance, that the degree of nutritiousness of any vegetable substance is generally proportionate to the quantity of azotised matter, which it contains. Now, as this is most abundant in the Cereal grains, we at once perceive why it is that these form the staple article of food of man in all countries. All the varieties of corn contain a considerable portion of Gluten and Albumen (the two most highly azotised vegetable products), and but a very small quantity of Gum, Sugar, or Starch—substances which contain little or no nitrogen. Keeping this fact in view, the following tables, given by Mr. *Johnston,* are interesting in a double point of view, and suggest some very useful hints with respect to diet and regimen.

"If we suppose," says he, "an acre of land to yield the following quantities of the usually cultivated crops, namely—

| | | | | |
|---|---|---|---|---|
| Of wheat, | 25 | bushels, | or | 1500 lbs. |
| Of barley, | 38 | .. | or | 2000 .. |
| Of oats, | 50 | .. | or | 2250 .. |
| Of pease, | 15 | .. | or | 1000 .. |
| Of beans, | 25 | .. | or | 1600 .. |
| Of Indian corn, | 60 | .. | or | 3120 .. |
| Of potatoes, | 10 | tons, | or | 22400 .. |
| Of turnips, | 25 | .. | or | 56000 .. |

The weight of dry starch, gluten, sugar, and gum, reaped in each crop, will be represented very nearly by the following numbers:—

| | Starch. | Gluten and Albumen. | Sugar and Gum. | Woody Fibre. |
|---|---|---|---|---|
| Wheat, . . . . | 825 lbs. | 315 lbs. | 60 | .. |
| Barley, . . . . | 1200 | 120 | 160 | .. |
| Oats, . . . . . | 1215 | 100 | 250 | .. |
| Pease, . . . . . | 420 | 260 | 20 | .. |
| Beans, . . . . | 670 | 370 | .. | .. |
| Indian corn, . . . | 2100 | 280 | 90 | .. |
| Potatoes, . . . . | 2688 | 224 | .. | 1253 |
| Turnip, . . . . | 3900 | 1400 | 5000 | .. |

If these crops are fair average returns from the same kind of land—if the acre, for example, which produces 25 bushels of wheat, will also produce 10 tons of potatoes and so on—we see that the crop of barley or oats will yield half as much again of Starch, and more than double the quantity of Sugar and Gum that a crop of wheat does; and that the quantity of these substances from the crop of turnips is five times as much of the one, and more than six times as much of the other. Again, if we compare the quantity of the Gluten derivable from the different crops, we find that nearly four times as much may be had from the turnip crop as from the wheat, beans, and Indian corn crops; and that the crops of barley and oats yield by much the smallest quantity of it.

It thus appears that, whether the nutritious property of vegetable food depends upon its Starch or its Gluten, a turnip crop is by far the most useful one that can be raised. Could this vegetable be rendered, says Mr. *Johnston*, an agreeable article of general human consumption, the produce of the land might be made to sustain a much larger population than under any of the other kinds of cropping alluded to.

However this may be, the table, we have given, sufficiently explains the reason of wheaten flour being so much more nutritious than barley or oaten flour—the greater abundance of Gluten in the one than in the other. One of the most striking physical peculiarities of this vegetable substance is the tendency which it shews, when moistened and exposed to the air, of undergoing changes similar to those of the putrefaction of animal bodies. This is owing to the circumstance of its containing Nitrogen as one of its component elements—in the proportion, in the case of wheat flour, of from 12 to 20 per cent.

The ultimate composition of vegetable gluten is remarkably analogous to that of the curd of milk, of the white of egg, and also of animal gelatine and of fibrine: hence it has often been called an Animo-Vegetable matter.

All these substances consist of nearly the same proportions of carbon, hydrogen, oxygen, and nitrogen, and they all contain, moreover, a small portion of phosphorus and sulphur. The cause therefore of the offensive odour, which they emit during decay, is sufficiently obvious.

In estimating, however, the nutritious properties of different kinds of food, we should not be guided by any single consideration in respect of its constitution—as for example by the mere amount of the Gluten of which it consists and of the Nitrogen which it contains—but rather pay due attention to all the various elements, of which it is made up. We should remember that the food has to serve not only to repair the great waste of the solid parts of the body, arising from the unceasing molecular absorption that is going on at every point, but also to supply the carbon that is continually exhaling from the lungs, during the process of respiration. Hence it is that the use of a diet consisting only of such articles as abound in one particular element, however nourishing this may be, cannot be long continued, without injury to health. The following observations of Mr. *Johnston* on this subject deserve to be generally known.

"The reader, therefore, will understand why a diet, which will keep up the human strength, is easiest compounded of a mixture of vegetable and animal food. It is not merely that such a mixture is more agreeable to the palate, or even that it is absolutely necessary,—for, as already observed, the strength may be fully maintained by vegetable food alone;—it is, because without animal food in one form or another, so large a bulk of vegetable food must be consumed in order to supply the requisite quantity of nitrogen in the form of gluten. Of ordinary wheaten bread alone, about 3 lbs. daily must be eaten to supply the nitrogen,* and there would then be a considerable waste of carbon in the form of starch, by which the stomach would be overloaded, and which, not being worked up by respiration, would pass off in excretions. The wants of the body would be equally supplied, and with more ease, by 1¾ lb. of bread, and 4 ounces of cheese.

"Of rice, again, no less than 4 lbs. daily would be required to impart to the system the required proportion of gluten; and it is a familiar observation of those who have been in India and other countries where rice is the usual food of the people, that the degree to which the natives distend, and apparently overload their stomachs with this grain, is quite extraordinary.

"The stomachs and other digestive apparatus of our domestic animals are of larger dimensions, and they are able, therefore, to contain with ease as much vegetable food, of almost any wholesome variety, as will supply them with the quantity of nitrogen they may require. Yet every feeder of stock knows that the addition of a small portion of oil-cake, a substance rich in nitrogen, will not only fatten an animal more speedily, but will also save a large *bulk* of other kinds of food." 237.

But, besides a due supply of the elements now mentioned, the food of all animals must contain a certain quantity of those Inorganic or Mineral materials which are found to exist in every part and tissue of the body, and which moreover are constantly passing out of the system in the various excretions. The blood and all the fluids contain a considerable proportion of saline matter of different kinds, sulphates, muriates, phosphates, and other compounds of Potash, Soda, Lime, and Magnesia; and every one knows that the solid strength of the bones is owing to the large quantity of earthy materials, which enter into their composition. Now the daily and hourly waste of all these substances must be replaced in some manner or another; else the health of the animal will be speedily observed to suffer. It is in the food consumed that we must look for this supply; and, if we examine the food of animals living in a state of nature, we shall always find that a very considerable quantity of saline matter enters into its constitution. In a former part of this article we have shewn that all plants contain more or less of alkaline and earthy salts—the proportion of these varying a good deal not only in different plants, but also according to the nature of the soil on which they grow. It is indeed a beautiful arrangement of Providence, as remarked by our author, that plants refuse to grow in a soil, from which they cannot readily obtain a due supply of soluble inorganic matter—since this very matter, which ministers first to their own wants, is afterwards surrendered by them to the animals they are destined to nourish.

By looking at the following table—which gives the proportionate quantities of the different inorganic substances in 100 lbs. of the ashes of four of the staple articles of vegetable food—we shall be able at once to perceive the relative fitness of each article to yield the necessary supply during

* "The flour being supposed to contain 15 per cent. of dry gluten, on which supposition all the above calculations are made."

the growth of the young animal, and to compensate for the excrementitious elimination that is going on from the body of the adult one.

| | Wheat. | Barley. | Oats. | Beans. |
|---|---|---|---|---|
| Potash .. .. .. .. | 19 | 12 | 6 | 19½ |
| Soda .. .. .. .. | 20½ | 12 | 5 | 38 |
| Lime .. .. .. .. | 8 | 4½ | 3 | 7¾ |
| Magnesia .. .. .. .. | 8 | 8 | 2½ | 7½ |
| Alumina .. .. .. .. | 2 | 1 | ½ | 1½ |
| Oxide of Iron .. .. .. | 0 | trace. | 1½ | 0 |
| Silica .. .. .. .. | 34 | 50 | 76½ | 6 |
| Sulphuric acid .. .. .. | 4 | 2½ | 1½ | 4 |
| Phosphoric acid .. .. .. | 3½ | 9 | 3 | 13¾ |
| Chlorine .. .. .. .. | 1 | 1 | ½ | 2 |
| | 100 | 100 | 100 | 100 |

It is chiefly from the various forms of the seeds of plants that herbivorous animals derive the Phosphate of Lime* which exists in most parts, (exclusively of its being the principal earthy constituent of the bones) and especially in the milk—to suit this for the food of the young creature during the early period of its life. The sugar of the milk supplies the comparatively small quantity of carbon necessary for its respiration, and the curd or *casein* yields not only the materials of the growing muscles and of the animal part of the bones, but also the phosphate of Lime required for the complete solidification of these pillars of the body. After giving an analysis of milk, Mr. *Johnston* shews how amply sufficient is the supply, which it yields for all the wants of the young offspring. He then presents us with a very interesting and instructive calculation:

"The quantity of solid matter thus yielded by the cow in her milk is really very large, if we look at the produce of an entire year. If the average yield of milk be 3000 quarts, or 750 gallons in a year—every 10 gallons of which contain bone-earth enough to form about 7 ounces of dry bone—then the milking of the cow alone exhausts her of the earthy ingredients of 33lbs. of dry bone. And this she draws necessarily from the soil!

"If this milk be consumed on the spot, then all returns again to the soil in the annual manuring of the land. Let it be carried for sale to a distance, or let it be converted into cheese and butter, and in this form exported, there will then be a yearly drain upon the land of the materials of bones, from this cause alone, equal to 30 lbs. of bone-dust. After the lapse of centuries, it is conceivable that old pasture lands, in cheese and dairy countries, should become poor in the

* It has been found that dogs, when fed upon fine white bread, did not live beyond 50 days or so; while others fed on household bread, in which there is a portion of the bran—that contains a larger proportion of earthy matter than the substance of the grain itself—continued to thrive for a much longer period of time. Such a fact as this alone is sufficient to prove the needlessness and cruelty of trying to discover the relative nutritiousness of different articles of food, by feeding animals with them exclusively.

materials of bones—and that in such districts, as now in Cheshire, the application of bone-dust should entirely alter the character of the grasses, and renovate the old pastures.

"Thus, as was stated at the commencement of the present section, the study of the nature and functions of the food of animals throws additional light upon the nature also and final uses of the food of plants. It even teaches us what to look for in the soil—what a fertile soil *must* contain that it may grow nourishing food—what we must add to the soil when chemical analysis fails to detect its actual presence, or when the food it produces is unable to supply all that the animal requires." 243.

We have said enough, we trust, to have excited a desire, in most of our readers, to know more of the very interesting subject of Dietetic and Agricultural Chemistry. Let them diligently peruse, nay study, the admirable work of *Liebig*—a man who, it seems to us, has produced as great a revolution in his science as *Black* and *Lavoisier* did at the close of last century. Those, who wish to have a lucid summary of the subject, will do well to purchase the little volume of Mr. *Johnston*; it is full of interesting matter, and withal very reasonable in price.

